# Covert Genetic Selections to Optimize Phenotypes

**DOI:** 10.1371/journal.pone.0001200

**Published:** 2007-11-21

**Authors:** Di Wu, Elizabeth Townsley, Alan Michael Tartakoff

**Affiliations:** 1 Monash Institute of Medical Research, Monash University, Monash Medical Centre, Melbourne, Victoria, Australia; 2 Department of Pathology, Cell Biology Program, Case Western Reserve University School of Medicine, Cleveland, Ohio, United States of America; University of Florida, United States of America

## Abstract

In many high complexity systems (cells, organisms, institutions, societies, economies, etc.), it is unclear which components should be regulated to affect overall performance. To identify and prioritize molecular targets which impact cellular phenotypes, we have developed a selection procedure (“SPI”–single promoting/inhibiting target identification) which monitors the abundance of ectopic cDNAs. We have used this approach to identify growth regulators. For this purpose, complex pools of *S. cerevisiae* cDNA transformants were established and we quantitated the evolution of the spectrum of cDNAs which was initially present. These data emphasized the importance of translation initiation and ER-Golgi traffic for growth. SPI provides functional insight into the stability of cellular phenotypes under circumstances in which established genetic approaches cannot be implemented. It provides a functional “synthetic genetic signature” for each state of the cell (i.e. genotype and environment) by surveying complex genetic libraries, and does not require specialized arrays of cDNAs/shRNAs, deletion strains, direct assessment of clonal growth or even a conditional phenotype. Moreover, it establishes a hierarchy of importance of those targets which can contribute, either positively or negatively, to modify the prevailing phenotype. Extensions of these proof-of-principle experiments to other cell types should provide a novel and powerful approach to analyze multiple aspects of the basic biology of yeast and animal cells as well as clinically-relevant issues.

## Introduction

Cell growth, migration, and ability to resist stress and infection depend on many factors which have been the subject of focused investigations. Further elucidation of these known determinants is of intrinsic interest and could lead to the design of corresponding molecular therapies. Nevertheless, when multiple components are involved–only some of which are known-it generally is unclear which should be targeted in order to have maximal effects on cellular performance. This optimization task becomes especially daunting when the goal is to manipulate performance–not with cells in culture–but in the full complexity of the animal. Moreover, the task of identifying growth inhibitory factors from high complexity cDNA or shRNA libraries is problematic since these are the components which disappear and therefore can be identified only by implementation of specialized strategies, e.g. [1], [2], [3], [4], [5].

The search procedure described below (SPI) starts with a complex library and selects–in one step and without requiring assays which depend on clonal growth-single cDNAs which can have positive or negative impact on growth or on other complex phenotypes. It thus identifies groups of “contributory” cDNAs, and establishes an approximate hierarchy of their importance. Moreover, it makes possible the identification of relevant genes without requiring that they be able to overtly correct a given phenotype. These features of SPI distinguish it from most classical cloning strategies.

**Figure 1:**
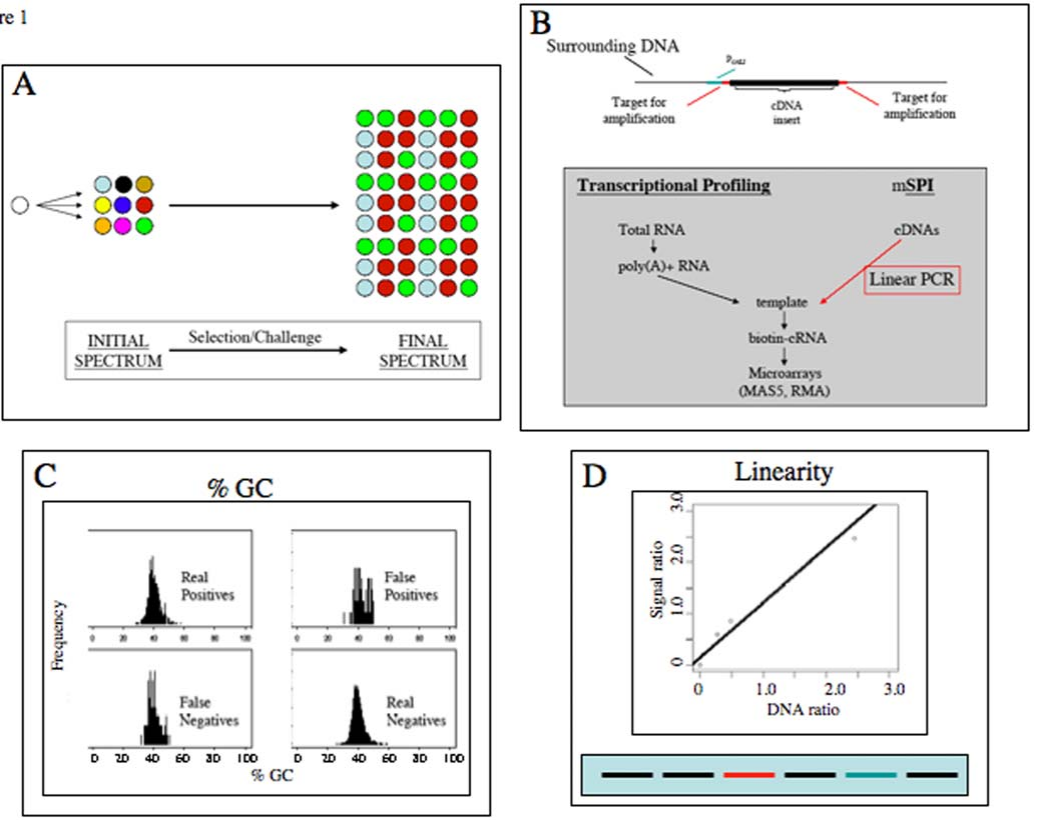
Overview A: Covert Selection Strategy Each color symbolizes the presence of a distinct ectopic cDNA. Significant changes occur when the cells are grown. cDNAs which sensitize cells to the culture conditions are expected to become depleted, while those which promote growth are expected to become enriched. B: cDNA Quantitation Upper: A typical yeast plasmid for cDNA expression, indicating the flanking targets (red) for PCR amplification. Lower: Comparison of transcriptional profiling to SPI. Both procedures generate biotinylated cRNAs using T7 RNA polymerase. In SPI, 30 cycles with the reverse primer are designed to yield linear single-stranded products, which are converted into a double-stranded copy in a single final step upon addition of the forward primer. C: GC Content of Subsets of cDNAs Having determined which of 933 cDNAs are detected on the microarrays, we subdivide the group into true positives, false positives, true negatives and false negatives. As shown, each group has nearly the same GC content. False negatives therefore are not due to difficulties in copying sequences of high GC content. D: Proportionality of Readout Two groups of 1000 strains were mixed in different proportions and then analyzed. The mean signal ratio between the groups is a near linear function of the relative DNA input, with a Pearson Correlation Coefficient of 0.986. The rectangular insert at the bottom of the figure represents the strategy of mixing different proportions of two pools of transformants.

To exemplify this approach–which could be extended to shRNAs and to animal cells using viral libraries at low multiplicity-we have used a bank of yeast transformants which differ from each other with regard to single ectopic cDNAs. We have then quantitated the abundance of each cDNA/transformant as the pool of cells grows in liquid culture (Figure 1A). The central premise is that cDNAs which promote growth or survival will cause the corresponding cells and their cDNAs to become more abundant, while those which impair growth will have inverse effects. The selection is “covert,” since the critical data pertain to relative abundance of cDNAs over time rather than to gross alteration of phenotype.

By identifying the universe of enriched and depleted cDNAs which are characteristic of a given cell, SPI establishes a signature of positive and negative synthetic genetic relations, which is the equivalent of what a mathematician would refer to as a “sensitivity analysis.” Such information will allow inroads into the understanding of seemingly asymptomatic knockout cells. Moreover, adaptations of SPI should provide novel access to optimization in the context of malignant growth, cell migration and susceptibility to infection.

SPI can be effective without requiring panels of deletion strains, “bar-coded” strains or plasmids, or further design of novel expression systems, e.g. [6], [7]. Since it is also likely to be able to make use of conventional cDNA libraries, it should be able to encompass the full diversity of splice variants of transcripts, e.g. for investigation of animal cells. The present study–in addition to identifying cDNAs which govern growth of yeast-provides a proof of principle for such undertakings.

## Results

### Preliminaries

The pool of yeast strains which we have studied carries a defined synthetic library of 5885 plasmids in which each cDNA is under control of a galactose-inducible promoter [8], [9]. The availability of these constructs made it possible to generate an approximately uniform mixture of corresponding transformants without induction, and then to test the impact of their expression when galactose was added. To develop procedures for plasmid recovery, copying of cDNA inserts, and probing of microarrays, we have first worked with small subsets of transformants.

The cDNA inserts are all present in a constant context in the library plasmids. To copy them without introducing the length and abundance bias which is characteristic of conventional PCR, we developed an efficient extraction procedure (Figure S1A) and a linear PCR procedure (“ds-Linear PCR”) (Figure 1B, S1B, S2) which in a final step generates double-stranded products. The rest of the procedure flows directly into the established chemistry, microarray probing with biotinylated cRNAs, and analytic algorithms developed for transcriptional profiling.

By processing a pooled subset of 933 defined transformants (before growth), we were able to tabulate the number of loci for which microarray signals were both expected and were detected (true positives), loci for which signals were expected but were not detected (false negatives), loci for which signals were not expected but were detected (false positives), and loci for which signals were not expected and were not detected (true negatives). In this situation, there were 94% real positives and 6% false negatives. We tabulated the GC content of cDNAs in each in these four categories and observe that false negatives were not enriched in cDNAs of high CG content, as might have been expected if they were difficult to copy-Figure 1C–and there was only modest depletion of longer cDNAs.

When DNA was recovered from two distinct pools of ∼1000 defined transformants (before growth) and different proportions were mixed and processed, the ratio of the mean signal values for each pool was nearly proportional to its relative abundance over the range examined–Figure 1D. In biological experiments, a two-fold change in relative signal intensity therefore corresponds to a comparable relative change in cDNA input.

We have inquired whether the pool of 5885 transformants retains its initial cDNA spectrum (i.e. the distribution of fluorescent intensities among all cDNAs) in liquid culture in selective medium under several conditions. Those which change to the greatest extent–the tails of the distributions–are referred to as “SPI Extremes.”

**Figure 2:**
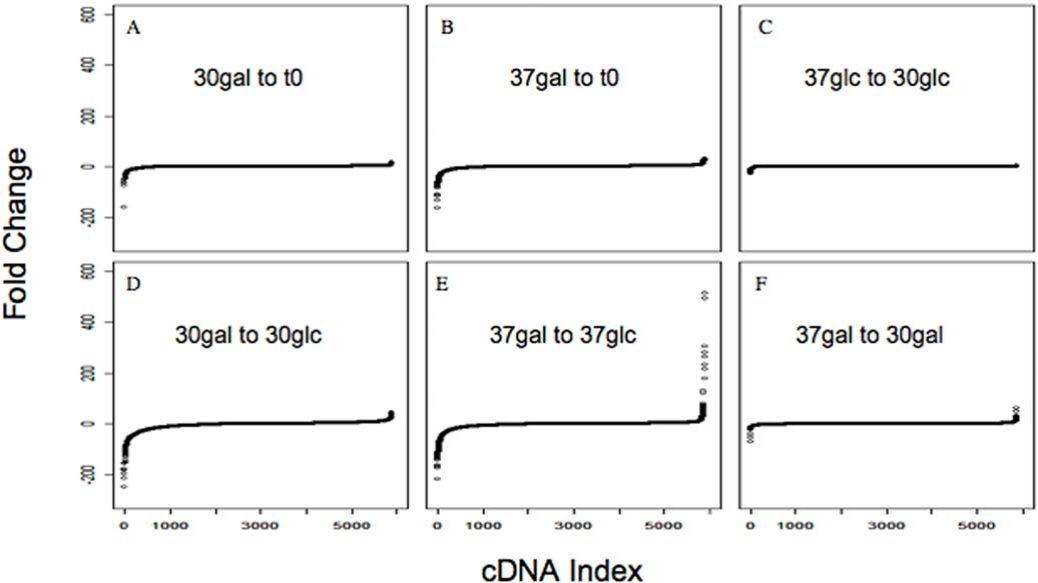
Differential cDNA Enrichment and Depletion (Fold Change) Upon Growth Replicate data sets were used to calculate fold-enrichment for six comparisons (see Methods). In each case, the horizontal axis (gene index) displays all cDNAs in order of their relative fold change. A: Cells grown at 30°C in glucose vs the initial sample. B: Cells grown at 37°C in glucose vs the initial sample. C: Fold-changes (B) divided by fold-changes (A). D: Cells grown at 30°C in galactose vs 30°C in glucose. E: Cells grown at 37°C in galactose vs 37°C in glucose. F: Fold-changes (E) divided by fold-changes (D).

When quadruplicate cultures were maintained for 30 generations at 30°C without induction (i.e. in glucose medium), there was little change of relative abundance of most cDNAs; however, some SPI Extremes could already be identified, with negative changes being obvious. Increases appear very modest (Figure 2A), in part because the algorithm which was used for these calculations places upper (and lower) limits on the numerical estimates. Figures 2B and 2C show that the spectrum which persisted at 37°C in glucose medium was nearly identical to that which persisted at 30°C in glucose medium. (Full data sets for all cDNAs are provided in Table S1, S2, S3, S4, S5 and S6.)

### Analysis of Growth

To identify cDNAs which affect growth upon deliberate overexpression, we have cultured samples of the full mixture of 5885 transformants in galactose medium for 30 generations at 30°C and compared their cDNA spectra to that of cells cultured in glucose medium-Figure 2D. Small numbers of cDNAs showed distinct differential enrichment or depletion (Table 1 and 2). Their identity is discussed below. For the 100 most enriched or depleted cDNAs, the quantitative estimates of fold change have a normalized standard deviation of ∼10% (see Methods). Although SPI can identify growth inhibitory cDNAs, there is little reason to expect functional coherence within this group since interference with many functions can be detrimental. No candidates are obviously related to plasmid maintenance.

**Table 1:** cDNAs Among the Top 100 at Both Tempratures

Standard Name	logFC 30°C	logFC 37°C	Comment
AKR2	5.4	7.0	Palmitoyl transferase ?
BET2	4	6.3	ER-Golgi transport
BNA1	4.4	5.2	Nicotinic acid biosynthesis
CTS2	3.9	3.8	chitinase ?
EGD2	4	4.5	NAC complex
GLS2	4.5	5.1	ER glucosidase
FAR8	3.8	4.5	pheromone response
FUN12	4.7	4.5	Translation initiation
GAL80	4.4	4.3	galactose repression
KEL3	4.7	5.5	?
LSM3	4.2	5.4	mRNA decay
MET18	4.3	4.2	DNA repair, transcription
MLC2	3.9	4.5	myosin light chain
MSH3	4	3.7	mismatch repair
NAS2	5.3	4.3	?
RPL5	4.4	6.9	Large ribosomal subunit assembly
RPS9A	5.1	8.1	Translational fidelity
SFB2	4.5	4.3	ER-Golgi transport
SLM1	5.5	6.2	Actin stress response
SOK1	4.6	4.7	cAMP signaling
SSU1	4.1	5.9	Sulfite efflux
SVS1	4.6	8.0	Vanadate resistance
TFS1	4.4	4.3	PKA signaling
THI80	4.7	7.5	Thiaminepyrophosphate-phosphokinase
UTP10	4.4	5.9	U3 snoRNA complex
VPS8	4.4	4.6	endosome
YPD1	4.8	4.7	osmotic sensor
YSP1	4.7	5.4	mitochondrial
ZDS2	4.3	5.2	telomeric silencing

**Table 2:** cDNAs Among the Bottom 100 at Both Temperatures

Standard Name	logFC 30°C	logFC 37°C	Comment
AGA1	−6.2	−6.7	agglutinin subunit
CAF4	−6.6	−5.8	mitochondrial fission
CCT6	−6.1	−5.8	actin, tubulin chaperonin
CCT7	−6.3	−7.0	actin, tubulin chaperonin
COS8	−6.9	−6.2	nuclear membrane
CRC1	−6.4	−6.9	mitochondrial transporter
CTS1	−7.5	−6.1	chitinase
DAL4	−6.1	−6.5	allantoin permease
DEP1	−5.9	−7.0	transcription
DHH1	−6.3	−6.1	mRNA decapping
ECM22	−6.0	−6.2	SREB protein
ENT1	−7.9	−6.8	endocytosis
FPS1	−6.4	−5.8	glycerol channel
MEP3	−6.7	−6.0	ammonium permease
MNN2	−6.6	−6.3	Golgi mannose transferase
NPL3	−6.2	−6.1	mRNA export
PAF1	−7.2	−6.0	transcription
PEX10	−7.2	−6.6	peroxisome biogenesis
PIB2	−6.2	−6.5	telomeric repression
PIG1	−6.1	−6.5	protein phosphatase
PXL1	−6.1	−6.3	polarized growth
SEC12	−6.2	−6.8	ER export
TRM1	−6.8	−6.2	tRNA methyl transferase
UBX7	−6.3	−6.2	binds Cdc48p
ZRG17	−7.7	−6.1	ER

By comparing data sets for cells cultured in galactose medium *vs* glucose medium, we minimized the possible contribution to SPI Extremes of host cell mutations which affect growth. To validate the impact of single SPI Extreme cDNAs we have used three growth tests at 30°C, with the understanding that the SPI protocol of growth for 30 generations greatly amplifies differences which are expected to be only modest after briefer growth intervals.

**Figure 3:**
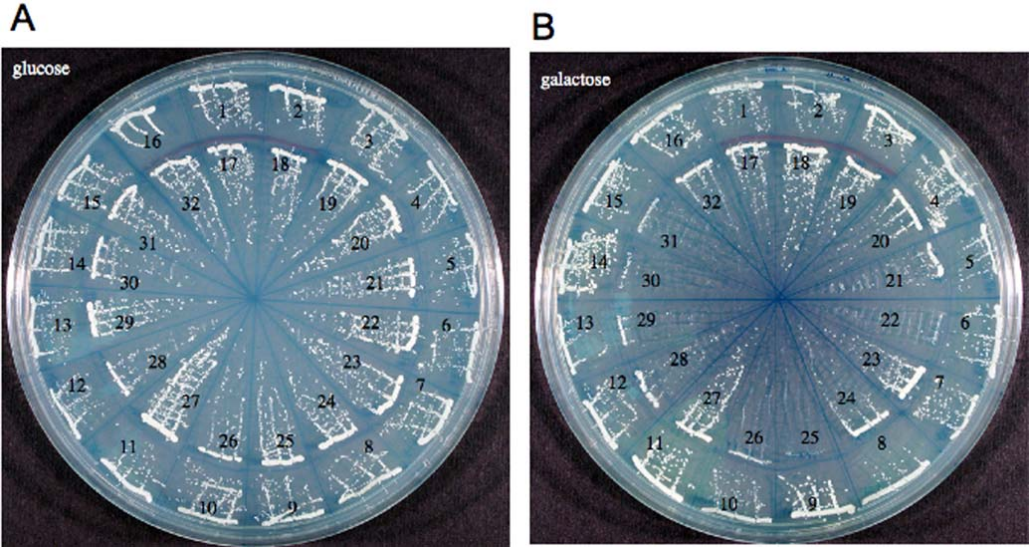
Validations on Solid Media Five control strains (#1–3, 18–19) which show little or no SPI enrichment/depletion and 27 SPI extreme strains including representatives from Table 3 were streaked on glucose dropout plates (Glc: A) or galactose dropout plates (Gal: B) and allowed to grow at 30°C. Strains which show SPI depletion are in the inner circle, while strains which show enrichment are in the outer circle. Growth of all strains is comparable on the glucose plates. On the galactose plate, the colony sizes of the strains often parallels expectations, both for increases and decreases. Among those illustrated, the concordance is modest, but more extensive surveys raise it to ∼80% (see text). The strains which do not conform to expectations could reflect differences between the requirements for growth in liquid vs solid media. The identities of the strains are given in Table 3. Strains #1–3 and #18–19 are controls whose SPI values range from −0.6 to 1.19

In the first test, we evaluated growth on solid media including galactose *vs* glucose (e.g. Figure 3A/B). All transformants gave colonies of comparable size on glucose plates. Of 70 strains which showed SPI depletion at 30°C less than −10 or SPI enrichment greater than +10, ∼80% were confirmed as being stimulatory or inhibitory by these plate tests (In Figure 3B, 9/13 of the transformants which showed SPI depletion were smaller than the average of the five controls, while 5/13 of the transformants which showed SPI enrichment generated colonies larger than the average size of the controls.) None showed growth characteristics on plates opposite those which are registered by SPI fold-change calculations.

The second test involved growth over 24 hr in liquid galactose *vs* glucose medium. This test appeared to be more sensitive for detection of the inhibitory cDNAs than the plate test, in that 11/13 of the strains in Figure 3 which showed SPI depletion grew more slowly in liquid culture than the controls. The normalized growth rate of the entire group of 13 strains was 0.57+/−0.15–Table 3. The liquid test appears less sensitive than the plate test for transformants which had consistently shown SPI enrichment. We observe, for example, a normalized growth rate of 0.93+/−0.12 for a set of 22 such strains (Table 3), i.e. comparable to the controls. As illustrated in the enrichment plot in Figure 2D, the positive SPI extremes at 30°C were less pronounced than for the depletions.

**Table 3:** Validation Tests

Gene Name	Systematic Name	Position in Figure 3	Colony Size *vs* SPI estimate	Liquid Growth Rate (normalized)	SPI Enrichment (logFC)	Sopko *et al.* Inhibitory Index
**TRANSFORMANTS ON THE PLATE IN ** **FIGURE 3**						
PLATE CONTROLS						
DRS2	YAL026C	#1 control		1.33	0.67	NT
MET1	YKR069W	#2 control		0.9	0.46	NT
STO1/CBC1	YMR125W	#3 control		1	−0.6	NT
GTT3	YEL017W	#18 control		0.94	1.19	NT
RBK1	YCR036W	#19 control		0.78	0.29	NT
OTHER TRANSFORMANTS ON THE PLATE (FIGURE 3)						
VID22	YLR373C	#4		1.15	3.6	NT
YJR039W	YJR039W	#5		0.92	4.4	NT
BNA1	YJR025C	#6		0.9	4.4	NT
MET18	YIL128W	#7		1.03	4.3	NT
YLR177W	YLR177W	#8		0.9	3.4	NT
YPD1	YDL235C	#9	agree	0.81	4.8	NT
BET2	YPR176C	#10		0.93	4	NT
NAS2	YIL007C	#11	agree	1.01	5.3	2
NRK1	YNL129W	#12		0.91	3.6	NT
GAL80	YML051W	#13		0.94	4.4	2
TFS1	YLR178C	#14	agree	0.84	4.4	NT
EGD2	YHR193C	#15	agree	1.09	4	NT
MAL31	YBR298C	#16	agree	1	3.5	NT
DOA1	YKL213C	#17		1.05	−6.4	NT
ZRG17	YNR039C	#20		0.87	−7.7	NT
GTS1	YGL181W	#21	agree	0.55	−3.4	NT
NPL3	YDR432W	#22	agree	0.49	−6.2	1
SMA1	YPL027W	#23		0.59	−2.9	NT
RTN2	YDL204W	#24		0.61	−4.5	NT
YTM1	YOR272W	#25	agree	0.56	−2.4	NT
YMR181C	YMR181C	#26	agree	0.52	−1.5	NT
ERG6	YML008C	#27		0.96	−5.9	2.5
CTR3	YLR411W	#28	agree	0.5	−3.8	NT
GTT1	YIR038C	#29	agree	0.55	−4.1	NT
DJP1	YIR004W	#30	agree	0.43	−5.4	1
GIP2	YER054C	#31	agree	0.52	−5.1	2.5
YDR161W	YDR161W	#32	agree	0.61	−4.3	NT
**OTHER TRANSFORMANTS USED FOR LIQUID GROWTH TESTS**						
TRANSFORMANTS WITH SPI ENRICHMENT						
FUN12	YAL035W			0.93	4.7	NT
LSM3	YLR483C-A				4.2	2.5
MLC2	YPR188C			0.68	3.9	NT
OYE2	YHR179W			0.79	1.9	NT
ROT2	YBR229C			0.89	4.5	NT
RPL5/L1	YPL131W			0.71	4.4	NT
RPS9A	YPL081W			1.03	1.9	NT
RPS9B	YBR189W			0.93	4.2	NT
SLM1	YIL105C			1.12	5.5	NT
UTP10 ?	YJL109C ?			0.9	4.4	NT
TRANSFORMANTS WITH SPI DEPLETION						
MPH3	YJR160C			0.5	−6.5	3.5
PTM1	YKL039W			0.57	−6.5	2
RPN4	YDL020C			0.51	−6.9	1.5
SEC4	YFL005W			0.51	−6.8	3
TRM1	YDR120C			0.51	−6.8	2
UBX7	YBR273C			0.29	−6.3	3
UPC2	YDR213W			0.56	−6.4	2.5
WTM2	YOR229W			0.51	−6.7	1
YDL211C	YDL211C			0.67	−6.4	1.5
YER064C	YER064C			0.47	−6.3	NT
YJL123C	YJL123C			0.34	−6.8	2
YML082W	YML082W			0.82	−6.2	3.5

In Sopko *et al*. 1 is the strongest inhibition. 4 is the least. NT: not tested.

In a final liquid medium validation test, we have mixed small pools of transformants, grown them in glucose or galactose medium and assessed the relative abundance of their cDNAs both at t_0_ and after ten generations, using ds-Linear PCR. As shown in Figure S3, those which had shown enrichment persisted, while those which had been depleted vanished.

Thus, the predictive power of SPI is high. SPI therefore could be applicable under circumstances in which clonal validation is not possible.

Since transformants can be cultured in galactose medium at temperatures up to 37°C without loss of viability (as judged by staining with FUN1), parallel experiments have also been conducted at this modestly stressful temperature (Figure 2E). Under these conditions, there were more examples of strong enrichment than at 30°C, while decreases remained modest. Table 1 lists those which are most enriched at both 30°C and at 37°C.

SPI can identify cDNAs which show differential enrichment upon exposure to stress. It therefore can also make predictions as to how to protect cells against stress or sensitize them to stress. For this purpose, we have evaluated the fold-enrichment of cDNAs after growth at 37°C *vs* 30°C in galactose–Figure 2F. Several groups of cDNAs were among the 100 which showed the highest differential enrichment (Table 4).

**Table 4:** cDNAs Differentially Enriched at 37°C

Mitochondrial	GPI-Related	Stress Responses	Protein Turnover
ACO1	CSH1	ECM10	PFA3
ATM1	GPI10	HSC82	RPN4
CEM1	GPI18	NTH1	VBA2
COX10	LCB5	PSR1	VMA2
COX15	TSC10		VPS30
ECM10	YPS3		VTC3
FMP40			
HEM1			
IDP1			
IML2			
MCT1			
MDL1			
MDM31			
MIA40			
MMM1			
MSR1			
PET54			
TAZ1			
TCM10			
YJL045W			
YOR022C			

## Discussion

Gene complementation strategies can be used to investigate diseases of monogenic origin but are seldom applied to diseases of more complex origin. The present study shows that related strategies can be extended to the analysis of both normal growth and growth at elevated temperature, providing an example of a complex phenotype. A fundamental difference between SPI and classical complementation cloning is that the goal is to identify a spectrum of “contributory genes” (or cDNAs), rather than a single entity (“command gene”), which by definition have graded impact on cellular phenotypes. Such genes are not identified by classical genetic strategies unless they overtly change the phenotype. A second fundamental difference is a loosening of the concept of genetic selection, since in SPI the selection is at the level of differential cDNA enrichment, rather than overt phenotypic correction. The two attendant characteristics of SPI which are integrally part of this strategy are the expression of single ectopic cDNAs, and the inclusive quantitative readout which is afforded by microarrays.

Two related plasmid-enrichment strategies have recently been described for yeast, both of which used microarrays and were based on two-way (two-color) comparisons. The first used a centromeric galactose-inducible cDNA library to investigate aspects of drug resistance. Plasmids were recovered and transcribed to yield single-stranded fluorescent probes which identified at least one logical target, but appeared also to generate non-specific products [10]. The second study used a high copy yeast genomic library to identify proteins which are related to the exosome. Plasmids recovered after growth were amplified through *E. coli* and then copied to yield fluorescent probes [11]. In neither study was attention given to the information content of depleted cDNAs.

The realization that multiple cDNAs can promote cell growth may seem at variance with the concept of there being a single rate-limiting-step. Nevertheless, such multiplicity is characteristic of optimization strategies for complex systems, reflecting the intricate interactions among numerous components which contribute to the sustenance of the whole. Unlike other complex systems, when working with cells the availability of gene libraries make it possible to learn which components are most critical. Clearly, the optimization strategy of SPI could be made more stringent by prolonging the growth/selection interval. Moreover, it could be iterated to identify dependent groups of functionally significant genes, e.g. by starting with a cell in which one SPI extreme cDNA is already overexpressed (or depleted).

Our control experiments (Figure 1) using defined mixtures of cDNA transformants show that SPI can yield data sets in which real positives overwhelmingly dominate the readout. Moreover, their recovery does not depend on GC content, the accuracy of replicates appears satisfactory (fold changes having a normalized standard deviation are ∼10%), and signal strength on the microarrays is a monotonic approximately linear function of input. Analysis of pools of transformants grown without induction already identified some differential depletion of cDNAs, presumably because unspecified factors were sequestered by the inserts.

Upon induction, considering that the library which we have used results in significant production of most of the proteins encoded by cDNAs [9], [12], it is notable that yeast tolerates overexpression of a large fraction of its genome.

To our knowledge, there has been no previous identification of single proteins which can increase the rate of growth or survival of yeast under standard growth conditions at 30°C. The cDNAs which become enriched could either directly accelerate the cell cycle or possibly bypass checkpoints or other events which delay rapid growth. Strikingly, twenty-nine cDNAs among the 100 which were most enriched at 30°C were also among the 100 most enriched at 37°C (Table 1). Several of these pertain to events which have previously been recognized as important control points: ribosome synthesis and translation initiation, for example, are generally considered to limit the speed of cell growth and are implicated in oncogenesis [13]. Moreover, censorship of glycoprotein exit from the ER is certainly needed for growth and expansion of the cell wall.

The six of these which encode proteins which are critical for protein synthesis or ribosome genesis-and therefore potentially affect the titer of many other proteins-are:

- Fun12p/eIF5B, a GTPase which functions in translation initiation by promoting binding of Met-tRNA_i_^Met^ to small ribosomal subunits and in subunit joining [14].
- Egd2p, a subunit of the NAC complex which binds nascent polypeptide chains and is thought to influence their delivery to the ER [15].
- Rps9A/B, a conserved small ribosomal subunit protein which is a major determinant of translational fidelity [16].
- Utp10p, a protein associated with U3 snoRNA which is required for 18S rRNA synthesis [17].
- Rpl5p/L1, which is required for assembly of large ribosomal subunits [18].
- Lsm3p, which functions in mRNA decay [19].

Three cDNAs encode proteins which are relevant to ER-Golgi transport:

- Gls2p/Rot2p, one of the subunits of the endoplasmic reticulum glucosidase II. This enzyme trims the N-glycans of newly-synthesized glycoproteins after their folding in the ER and prior to exit to the Golgi. It is also required for efficient ER-associated degradation [20], [21]. Suboptimal maturation and expression of one or more of its glycoprotein substrates–or perhaps a cell wall component such as 1,6-β-glucan–could normally limit cell growth.
- Bet2p, a prenyl transferase which is required to anchor Ypt1p and Sec4p, which function in ER-Golgi transport [22].
- Sfb2p, a probable component of COP II vesicles [23].

Two encode components which impact the actin cytoskeleton

- Slm1p, which regulates actin organization in response to stress [24].
- Mlc2p, the regulatory light chain of myosin I [25].

SPI readily identifies cDNAs which become depleted upon growth. As mentioned above, many of these can be validated qualitatively, both by following the colony size of single transformants on solid media (Figure 3) and by monitoring the growth of pure cultures in liquid medium (Table 3).

**Figure 4:**
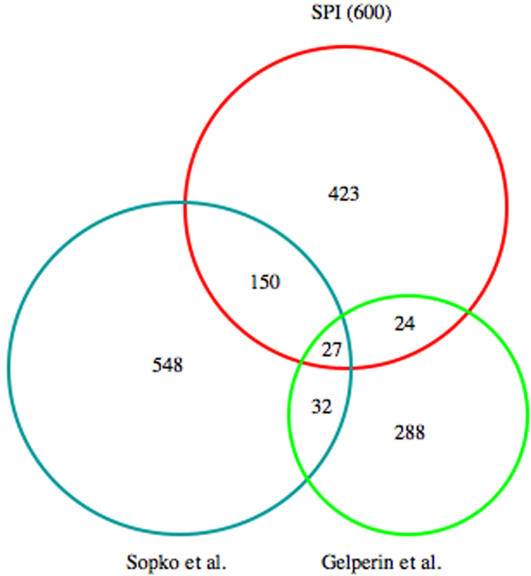
Venn Diagram comparison of data sets which have identified growth-inhibitory cDNAs. See Table 5 and 6 for detail.

Previous studies have used panels of *S. cerevisiae* transformants to evaluate the ability of individual cDNAs, GST fusions and gene fragments to inhibit growth (Table 5, Table S7) [8], [26], [27], [28], [29], [30], [31]. These mostly qualitative assays monitor clonal growth on solid media, which places distinct demands on cells (e.g. [32]), while SPI is based on a competition assay in liquid culture and lends itself to quantitative comparisons. Table 5 summarizes the vectors used and numbers of inhibitory cDNAs which were identified. Table 6 tabulates the extent of overlap with SPI. From the SPI data set we have used the 600 most depleted cDNAs for this comparison since the two recent large-scale studies based on colony growth of cDNA transformants have identified 454 and 759 inhibitory cDNAs, respectively [8], [12]. Judging from this comparison, SPI shows the greatest overlap with the one study which has used the same GST-ORF expression library that we have used [8]–although it uses a different host cell. The Venn diagram in Figure 4 illustrates the overlap between SPI and this study as well as a recent study which used an ORF-GST fusion library [12].

**Table 5:** Screens for Inhibitory cDNAs

Sequences	Design/Medium	Vector	# Toxic Loci	Reference
ORFs	Solid/Qualitative	*CEN/GAL1*	15/25,000	Liu *et al*
ORF	Solid/Qualitative	*2μ/GAL1*	24/10,000	Espinet *et al*
ORFs	Solid/Qualitative	*CEN/GAL*	185/180,000	Stevenson *et al*
ORF-GSTs	Solid/Qualitative galactose/glycerol/ethanol	*2μ /GAL*	371/5854	Gelperin *et al*
fragments	Solid/Qualitative	*2μ /TetO-CYC1*	454/84,000	Boyer *et al*
GST-ORFs	Solid/Semiquantitative	*2μ /GAL1*	757/5800	Sopko *et al*
GST-ORFs	Liquid/Quantitative	*2μ /GAL1*	2126/5885	SPI

In each of the indicated studies, large panels of cDNAs were tested. The number which were toxic is tabulated in the fourth column. The data set from Gelperin *et al*. is based on their growth experiments in the presence of galactose, glycerol and ethanol.

**Table 6:** Coincidence of Inhibitory cDNAs

SPI (600)	Sopko *et al.* (757)	Gelperin *et al.* (371) galactose/glycerol/ethanol	Boyer *et al.* (454)	Liu *et al.*	Espinet *et al.*	Stevenson *et al.*	No. Shared
x				x			1
x					x		1
x						x	15
x		x					51
x			x				63
x	x						177
	x	x					59
x	x	x					27
x	x	x	x				5

This Table tabulates the overlap between the SPI data (600 most depleted cDNAs) and the data sets from three other recent surveys. In each row, the x's indicate the data sets which are compared. Clearly the SPI data agree most closely with those of Sopko *et al.* A single cDNA overlaps between the studies of Boyer et al., Espinet *et al.*, *Liu et al.*, and Stevenson *et al*.: *NSR1*, but *NSR1* was not classified as growth inhibitory by Sopko *et al*. or in SPI.

Table 7 enumerates the 27 cDNAs which are shared by the SPI 600, the second study which used the same library, and the study which is based on expression of ORF-GST fusions [12]. Of this group, only 5 are also shared by the investigation which surveys random transcriptional fragments [27]. Proteins which affect growth can do so as part of complexes. Since the functionality of such complexes may be perturbed by alterations of their stoichiometry [33], we have asked whether the group of 27 depleted SPI extremes enumerated in Table 7 is enriched in proteins which are known to be in stable complexes. Judging from the database [http://portal.curagen.com/cgi-bin/com.curagen.portal.servlet.PortalYeastListmodeInList], there is no obvious enrichment. A similar conclusion has been reached in the second study which surveys the same GST-ORF library [8].

**Table 7:** Inhibitory cDNAs in Multiple Studies

PRP45	YAL032C
YBL009W	YBL009W
PET9	YBL030C
HEK2	YBL032W B
SEC17	YBL050W
SCO2	YBR024W
HEX3	YDL013W
PPH3	YDR075W
CCT6	YDR188W
NAB2	YGL122C
MDR1	YGR100W
STP2	YHR006W
YHR115C	YHR115C
YHR161C	YHR161C
YHR177W	YHR177W
DP1	YIR004W
YJL123C	YJL123C B
PAP1	YKR002W
SSK1	YLR006C B
PWP1	YLR196W B
ADY4	YLR227C
SFP1	YLR403W
MSC1	YML128C B
DSK2	YMR276W
PUS4	YNL292W
RVB2	YPL235W
YPR011C	YPR011C

The Table lists the cDNAs which are among the 600 most depleted group from the present study and are also classified as inhibitory by two other studies (Sopko *et al*., Gelperin *et al*.). The initial B designates those which were also found to be inhibitory by Boyer *et al*.

With regard to stress resistance, among the 100 which show the greatest differential enrichment at 37**°**C (*vs* 30°C) are 21 cDNAs which encode mitochondrial proteins, reminiscent of longstanding observations of the importance of functional mitochondria for survival at elevated temperature [34]. Additionally, six contribute to expression of GPI-anchored proteins, which concentrate at the cell surface [35], [36]. Table 4 also lists groups of differentially enriched cDNAs which are implicated in protein turnover or stress responses.

Calculations of differential enrichment at 37°C are clearly a composite, including cDNAs which were enriched at 37°C as well as those which were depleted at 30°C. Figure S4 separates out these two subsets by comparing the 37°C *vs* 30°C data to both the enrichment at 37°C and the depletion at 30°C. The subgroup encoding mitochondrial proteins accounts for ∼40% of those which show an increment at 37°C.

One might expect that the SPI Extreme (enriched) cDNAs would correspond to essential genes; however, as for the total genome, only about 20% of the cDNAs which are most enriched at 30°C (or 37°C) correspond to genes which are essential under standard growth conditions (*Saccharomyces* Genome Database). Thus, survival of the organism under laboratory conditions cannot require those genes which have the potential to be most beneficial. These “accessory” beneficial genes represent an evolutionary opportunity (or therapeutic opportunity) which can be detected by SPI.

It is also of interest to ask whether SPI Extreme enriched cDNAs correspond to mRNAs which are upregulated at 37°C (in either glucose or galactose medium). The comparison is greatly simplified since the same microarrays can be used for both purposes. As shown in Figure S5, there is minimal concordance. This discrepancy could signify that the normal circuitry of gene expression seldom allows the cell to manipulate the level of single transcripts, i.e. bystander transcripts which would be co-modulated would sabotage any attempt to up- or down-regulate those which–by themselves–could be most useful.

We expect that the greatest prospect for implementation of SPI will be in the context of animal cell biology, where it should again exemplify the utility of genetic approaches outside the normal realm of genetic inquiry. This is especially because of the difficulty of choosing optimal therapeutic targets for many diseases. In each case, subtle selective events are surely always at work. SPI makes it possible to use a covert selection “*in situ*” to identify the single genes which should be manipulated to influence such phenotypes.

## Materials and Methods

### Cells and cDNA Library

These materials were obtained from M. Snyder and D. Gelperin (Yale University) [9]. The haploid host cell was YC123 = SF657-2D = Snyder strain 258 (MATa *pep4-3 his4-580 ura3-52 leu2-3, 112*). The 10 kb pEG(KG) 2μ vector used for cDNA expression carried both *URA3* and *leu2-d* selectable markers and appends GST to the N-terminus of each product [37]. Frozen stocks of single transformants and pools of transformants were prepared by standard methods.

### Cell Growth, Plasmid and RNA Recovery

Liquid cultures were established from single colonies. After growth in uracil dropout medium at room temperature, aliquots were diluted to A600 = 0.1 using uracil drop-out glycerol-lactate medium (2% glycerol, 2% lactate, 0.05% glucose, 0.67% bacto-yeast nitrogen base without amino acids, pH 5.5), grown overnight at room temperature to A600 = 1−2 and then harvested by sedimentation.

5 ml samples of cells were washed with water, broken by vortexing with glass beads, and extracted with a Qiagen DNA extraction kit.

To evaluate cell viability, samples were stained with FUN1 (Molecular Probes (F-7030)) and examined by epifluorescence. Living cells showed bar-shaped orange structures in the vacuole while dead cells lacked this signal and were predominantly green.

To study growth at 30°C and 37°C, frozen pools including all 5885 strains were thawed, washed, and then adjusted to A600 = 0.05−0.1 in glycerol-lactate medium. 5 ml at OD600 = 1−2 was set aside and refrozen to provide a t_0_ sample. Duplicate cultures were supplemented with 2% glucose or 2% galactose and then shaken at 30 and 37°C. Growth was monitored at 600 nm and aliquots of each culture were rediluted to A600 = 0.05−0.1 so that the A600 never exceeded 1.5. Duplicate 5 ml cultures were snap frozen in liquid nitrogen after a total of 30 generations.

For RNA analysis, we used hot phenol [38] to extract logarithmic cultures growing in glycerol-lactate medium supplemented with 2% glucose- or 2% galactose and processed the samples in accordance with Affymetrix protocols.

### Ds-Linear PCR Amplification

Linear amplification of cDNA inserts was performed using a mixture of DNA polymerases (Taq and pfu) and the reverse primer (5′-TGTAATACGACTCACTATAGGGGATCCCCGGGAATTGCCATG-3) which includes the T7 phage RNA polymerase sequences (5 min pre-denaturation at 94°C, followed by 30 cycles of 1 min at 94°C, 1 min at 58°C and 7 min at 67°C, and concluding with 10 min at 70°C). The concluding step with addition of forward primer (5′-TGGTGGTGGTGGAATTCCAGCTGACCACC-3′) and Hotstart taq polymerase consisted of 6 min at 94°C, 5 sec at 95°C, 1 min at 58°C, 7 min at 67°C and 10 min at 70°C. The primers were complementary to sequences which flank the inserts and do not include the regions which encode GST or the GAL promoter.

### Microarray analysis

Ds-linear PCR products were pooled, concentrated and purified using a Qiagen column. In general, 200 ng of PCR product was used to generate biotinylated cRNA probes for Affymetrix S98 DNA microarrays. Samples were processed at the University Affymetrix facility, scanning the microarrays with a GeneArray scanner and preprocessing data using RMA (Robust multiarray average) [39], [40]. For background-correction, normalization, and signal intensity calculations. In all experiments described below we studied independent triplicates or quadruplicates and based analysis on those cDNAs for which signals are classified as “Present” in each sample, using the MAS5 algorithm. Normalized signals from totally independent replicates have a mean correlation coefficient of 0.963014.

To learn whether cDNAs which give only a weak signal at t = 0 can be studied reproducibly, we asked whether there is any correlation between initial signal intensity and the consistency of their presence (or absence) after 30 generations of growth. We did not detect any such correlation using Student's t-test. Signal intensity data from replicate samples were used to perform differential expression analysis by fitting a linear model (one way ANOVA) using the limma package. The P values were adjusted with an empirical Bayesian method and the Benjamini and Hochberg False Discover Rate. Fold changes of >2 with p<0.05 were considered meaningful. Average fold-changes were then used to produce the enrichment or “S-plots” and to identify the most enriched and most depleted cDNAs. Fractional values reflect depletion and are represented according to the following convention: 0.1 = −10; 0.2 = −5 etc. Standard deviations of the logarithm of fold change values were also estimated using the limma (Linear Models for Microarray Analysis) package [41]. For the groups of fold changes listed in Table S1, S2, S3, S4, S5 and S6, the means +/− standard deviation are −6.43+/−0.60 (30°C decreases); 4.24+/−0.48 (30°C increases); −6.24+/−0.47 (37°C depletion); 4.90+/−0.47 (37°C increases). Given the normalization procedures of RMA, the fold change estimates are relative.

### Validation

Growth of individual transformants was studied by streaking single colonies onto solid media (2% glycerol, 2% lactate in uracil drop-out medium, pH5.5+2% glucose or+0.05% raffinose and 2% galactose) at 30°C and following their growth for increasing periods of time. We also have monitored the growth of duplicate cultures of single transformants in liquid uracil drop-out glycerol-lactate medium supplemented with 2% glucose *vs* 2% galactose at 30°C over 24 hrs. Cultures were initiated with an A600 = 0.03. Light scattering at 620 nm was measured with a Coulter-Beckman plate reader as a function of time. Kinetic rate constants were estimated using Origin and the galactose data were compared to the rate of growth of parallel cultures of each strain in glucose medium. To provide a uniform point of comparison between experiments, the five control strains (#1–3 and #18–19 in Figure 3) were included in each experiment, their average rate of growth was normalized to 1.0 and individual strains were then compared to them.

Alternatively, mixtures of transformants were cultured in liquid media identical to those used for the initial experiment, processed using ds-Linear PCR and the resulting double-stranded DNA products were then resolved on Agarose gels.

To monitor growth in the absence of galactose, selected cDNAs (without the GST moiety) were copied by conventional PCR and subcloned into a *URA3* vector in which transcription was under control of a *MET25*, methionine-represssible, promoter [42]. Corresponding transformants were then tested on plates made with complete medium *vs* methionine-dropout medium.

## Supporting Information

Figure S1 Plasmid Extraction and Examples of ds-Linear PCR Products A)DNA was extracted from a set of six transformants, restricted with SmaI, and fractionated on a gel and stained with ethidium bromide. Note the progressively increasing sizes of the inserts, whose abundance is comparable to that of the endogenous 2-micron circle. B) An equivalent pool of eight plasmids was used for ds-Linear PCR. The fractionated products are illustrated adjacent to size standards (*)
(0.10 MB DOC)

Figure S2 Determination of the End-Point for ds-Linear PCR Figure S1 shows that the products of ds-Linear PCR yield sharp bands, which is surprising. To determine the approximate point of arrest of the linear cycles, the products of the first 30 cycles (30R)-which included only the reverse primer-were incubated with any of three forward primers for the final step of the reaction. Our conventional primer (F)-not shown-and one of the test primers (F1) caused an obvious increase of intensity of the product bands. A second test primer (F2) did not. The point of arrest is therefore between sites complementary to F1 and F2. Primer F2 is 103 base upstream of primer F1, which is 234 bases upstream of primer F. The sequence of F1 is 5′-ATGTGCCTGGATGCGTTCC-3′. The sequence of F2 is 5′-TGAAAATGTTCGAAGATCGTTTATGTC-3′.
(0.14 MB DOC)

Figure S3 Validation by PCR. A small pool of transformants (a–d) was either extracted at once or allowed to grow for ten generations at 30°C in glucose or galactose medium, before DNA extraction. Their degree of enrichment or depletion (fold-change) in the initial experiment with 5885 transformants is indicated in parentheses. Triplicate samples of the initial and final pools were copied by ds-Linear PCR and the products were analyzed. Note that the enriched cDNA becomes dominant and that those which had been depleted vanish, by comparison to a control. The control plasmid had become neither enriched nor depleted when part of the complete pool of strains in the initial experiment.
(0.18 MB TIF)

Figure S4 Analysis of Differential Enrichment (37°C vs 30°C) cDNAs which show strong differential enrichment in Table 2 can do so either because of enrichment at 37°C or depletion at 30°C. Correspondingly, cDNAs which show strong differential depletion at 37°C can do so either because of depletion at 37°C or enrichment at 30°C. The three panels to the left concern the 100 cDNAs which show the greatest relative depletion at 37°C, while those at the right concern the 100 which show the greatest enrichment. Note, in each case, the presence of cDNAs which show both types of behavior.
(0.05 MB TIF)

Figure S5 Comparison of 30°C SPI Data to Transcriptional Profiles. Panel A represents SPI second order fold data which are calculated by dividing the 37°C vs 30°C fold change in galactose by the 37°C vs 30°C fold change in glucose. In panels B–D, the second order SPI data are compared to RNA transcript profiles of the same host cell (without plasmid) cultured at 30°C or 37°C in glucose or galactose medium. In panel B the RNA signals at 37°C in glucose are compared to RNA data at 30°C in glucose. In panel C the RNA signals at 37°C in galactose are compared to 30°C in galactose. In panel D the (37°C galactose/30°C galactose) ratio is compared to the (37°C glucose/30°C glucose) ratio. As can be readily seen, there is no widespread correspondence between the levels of mRNAs and SPI data. The transcripts which do show strong induction upon addition of galactose include the familiar set of genes GAL1, GAL2, etc.
(0.07 MB TIF)

Table S1 glc30 vs control. For Tables S1, S2, S3, S4, S5 and S6: Full SPI Data Sets. The averaged data are divided into six groups as in Figure 2, comparing S1) Cells cultured in glucose medium at 30°C to t_0_ samples, S2) Cells cultured in glucose medium at 37°C to t_0_ samples, S3) Cells cultured in glucose medium at 37°C to cells cultured in glucose medium at 30°C, S4) Cells grown at 30°C in galactose vs 30°C in glucose, S5) Cells grown at 37°C in galactose vs 37°C in glucose, and S6) Cells grown at 37°C in galactose vs 30°C in galactose. The successive columns indicate the Affymetrix identification number interrogated, the Systematic and Gene names, the logarithm of fold change (FC), the t statistic (1/normalized standard deviation), the P value, adjusted P value, and B statistic.
(1.82 MB XLS)

Table S2 glc37 vs control
(1.82 MB XLS)

Table S3 gal30 vs glc30
(1.82 MB RTF)

Table S4 glc37 vs glc30
(1.82 MB XLS)

Table S5 gal37 vs glc37
(1.82 MB XLS)

Table S6 gal37 vs gal30
(1.82 MB XLS)

Table S7 Inhibitory cDNAs, Comparison to Previous Studies. The citations of earlier investigations which have identified yeast cDNAs and gene fragments which inhibit growth are included in the text. In the study of Sopko et al. the magnitude of growth inhibition is designated 1–4, with 1 being the strongest inhibition.
(0.28 MB XLS)
