# Supplementary figures and images for: Covert Genetic Selections to Optimize Phenotypes
Source: PLoS One. 2007 Nov 21;2(11):e1200. doi: 10.1371/journal.pone.0001200 (PMC2075469; doi:10.1371/journal.pone.0001200)

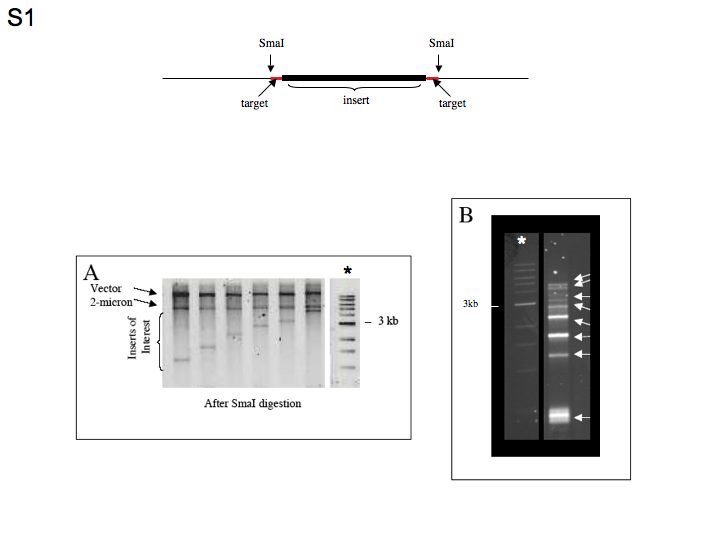

Supplement: Figure S1 — Plasmid Extraction and Examples of ds-Linear PCR Products A)DNA was extracted from a set of six transformants, restricted with SmaI, and fractionated on a gel and stained with ethidium bromide. Note the progressively increasing sizes of the inserts, whose abundance is comparable to that of the endogenous 2-micron circle. B) An equivalent pool of eight plasmids was used for ds-Linear PCR. The fractionated products are illustrated adjacent to size standards (*) (0.10 MB DOC) [file pone.0001200.s001.tif]

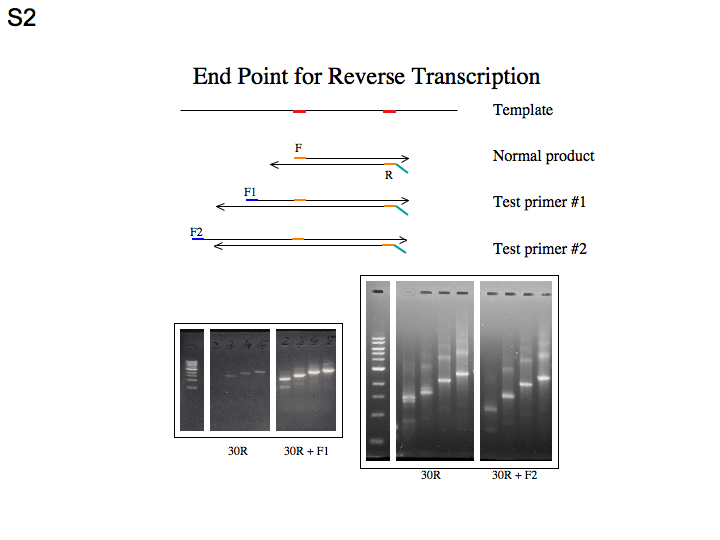

Supplement: Figure S2 — Determination of the End-Point for ds-Linear PCR Figure S1 shows that the products of ds-Linear PCR yield sharp bands, which is surprising. To determine the approximate point of arrest of the linear cycles, the products of the first 30 cycles (30R)-which included only the reverse primer-were incubated with any of three forward primers for the final step of the reaction. Our conventional primer (F)-not shown-and one of the test primers (F1) caused an obvious increase of intensity of the product bands. A second test primer (F2) did not. The point of arrest is therefore between sites complementary to F1 and F2. Primer F2 is 103 base upstream of primer F1, which is 234 bases upstream of primer F. The sequence of F1 is 5′-ATGTGCCTGGATGCGTTCC-3′. The sequence of F2 is 5′-TGAAAATGTTCGAAGATCGTTTATGTC-3′. (0.14 MB DOC) [file pone.0001200.s002.tif]

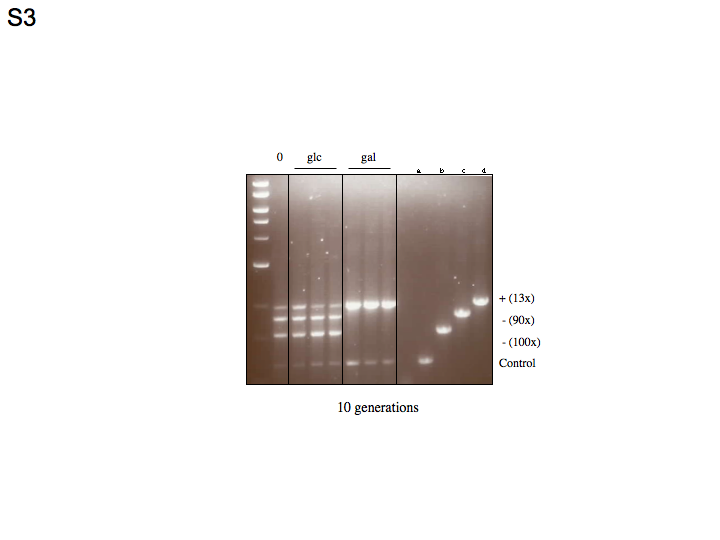

Supplement: Figure S3 — Validation by PCR. A small pool of transformants (a–d) was either extracted at once or allowed to grow for ten generations at 30°C in glucose or galactose medium, before DNA extraction. Their degree of enrichment or depletion (fold-change) in the initial experiment with 5885 transformants is indicated in parentheses. Triplicate samples of the initial and final pools were copied by ds-Linear PCR and the products were analyzed. Note that the enriched cDNA becomes dominant and that those which had been depleted vanish, by comparison to a control. The control plasmid had become neither enriched nor depleted when part of the complete pool of strains in the initial experiment. (0.18 MB TIF) [file pone.0001200.s003.tif]

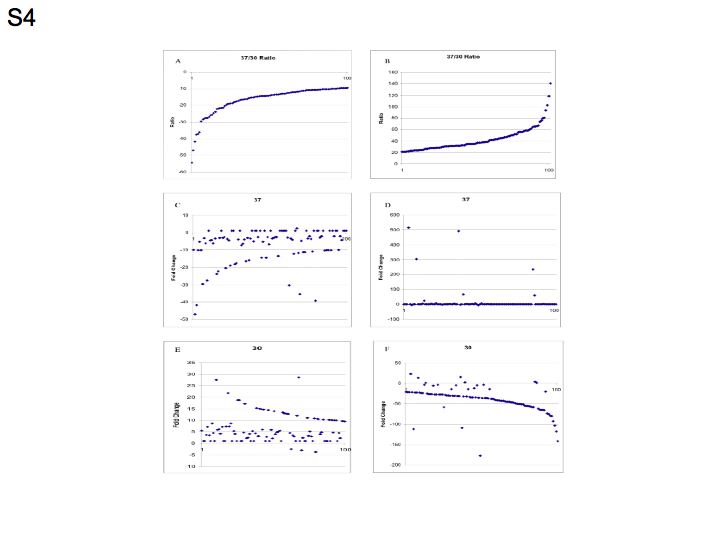

Supplement: Figure S4 — Analysis of Differential Enrichment (37°C vs 30°C) cDNAs which show strong differential enrichment in Table 2 can do so either because of enrichment at 37°C or depletion at 30°C. Correspondingly, cDNAs which show strong differential depletion at 37°C can do so either because of depletion at 37°C or enrichment at 30°C. The three panels to the left concern the 100 cDNAs which show the greatest relative depletion at 37°C, while those at the right concern the 100 which show the greatest enrichment. Note, in each case, the presence of cDNAs which show both types of behavior. (0.05 MB TIF) [file pone.0001200.s004.tif]

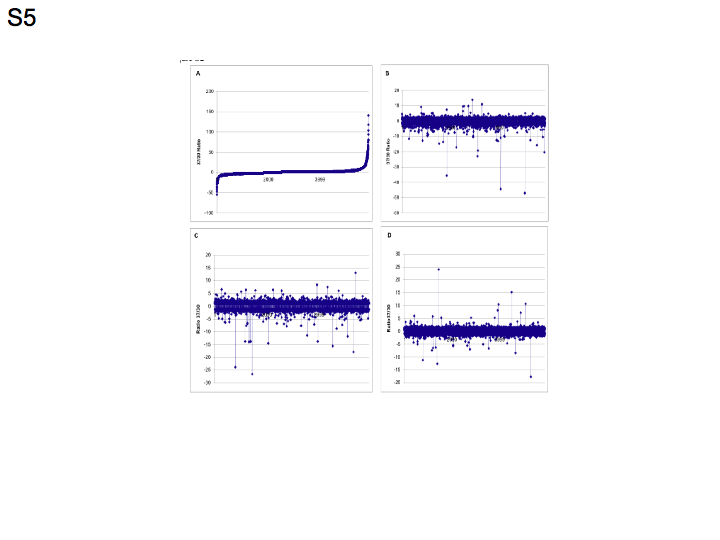

Supplement: Figure S5 — Comparison of 30°C SPI Data to Transcriptional Profiles. Panel A represents SPI second order fold data which are calculated by dividing the 37°C vs 30°C fold change in galactose by the 37°C vs 30°C fold change in glucose. In panels B–D, the second order SPI data are compared to RNA transcript profiles of the same host cell (without plasmid) cultured at 30°C or 37°C in glucose or galactose medium. In panel B the RNA signals at 37°C in glucose are compared to RNA data at 30°C in glucose. In panel C the RNA signals at 37°C in galactose are compared to 30°C in galactose. In panel D the (37°C galactose/30°C galactose) ratio is compared to the (37°C glucose/30°C glucose) ratio. As can be readily seen, there is no widespread correspondence between the levels of mRNAs and SPI data. The transcripts which do show strong induction upon addition of galactose include the familiar set of genes GAL1, GAL2, etc. (0.07 MB TIF) [file pone.0001200.s005.tif]
